# Hypovitaminosis D Influences the Clinical Presentation of Immune Thrombocytopenia in Children with Newly Diagnosed Disease

**DOI:** 10.3390/jcm8111861

**Published:** 2019-11-03

**Authors:** Davor Petrovic, Benjamin Benzon, Marijan Batinic, Srđana Culic, Jelena Roganovic, Josko Markic

**Affiliations:** 1 Department of Pediatrics, University Hospital of Split, Spinciceva 1, 21000 Split, Croatia; marijan.batinic23@hotmail.com (M.B.); srdjana.culic.sc@gmail.com (S.C.); jmarkic@mefst.hr (J.M.); 2 Department of Neuroscience, University of Split School of Medicine; Soltanska 2, 21000 Split, Croatia; benzon.benjamin@gmail.com; 3 Department of Pediatrics, University Hospital of Rijeka, Braće Branchetta 20/1, 51000 Rijeka, Croatia; roganovic.kbcri@gmail.com; 4 Department of Pediatrics, University of Split School of Medicine, Soltanska 2, 21000 Split, Croatia

**Keywords:** 25(OH)D, vitamin D deficiency, immune thrombocytopenia

## Abstract

Immune thrombocytopenia (ITP) is an acquired autoimmune disorder characterized by isolated thrombocytopenia defined as platelet count in peripheral blood <100 × 10^9^/L. Hypovitaminosis D is very common in children with autoimmune diseases. To analyze whether hypovitaminosis D is associated with the clinical presentation of ITP in children, medical records of 45 pediatric patients with newly diagnosed immune thrombocytopenia in the coastal region of Croatia were evaluated. The severity of bleeding was assessed using two bleeding scores. Children with lower 25-hydroxyvitamin D (25(OH)D) values had higher values of the skin-mucosa-organ-gradation (SMOG) bleeding score and respectively more severe bleeding on diagnosis of ITP. With further analysis of the main domains of that score, we found that patients with a lower 25(OH)D value had more severe bleeding in the skin and organs. When 25(OH)D and ITP Bleeding Scale (IBLS) score were analyzed, a negative correlation was found, but it was not significant. Our findings suggest that hypovitaminosis D influences the severity of the clinical presentation of ITP in children on initial diagnosis of the disease. Therefore, therapy with 25(OH)D could be a new potential option for treatment of ITP. To investigate the connection between 25(OH)D and the incidence and severity of ITP, further studies, especially randomized controlled studies, are needed.

## 1. Introduction

Immune thrombocytopenia (ITP) is an acquired autoimmune disorder characterized by isolated thrombocytopenia defined as platelet count in peripheral blood <100 × 10^9^/L [1]. In most patients, the number of platelets in peripheral blood is less than 30 × 10^9^/L—of which, 80% have a platelet count < 20 × 10^9^/L, and 44% have a count <10 × 10^9^/L [2,3]. 

The incidence of ITP in children is approximately 4–8 per 100,000 person-years [1,4,5]. It is usually a benign disease and spontaneously subsides within 3 months. ITP lasting for 3–12 months is called persistent thrombocytopenia, while thrombocytopenia lasting >12 months is considered as a chronic form of ITP (occurring approximately in 20% of cases) [6,7,8].

It is assumed that the key pathophysiological mechanism important for ITP development is a loss of immune self-tolerance followed by the development of autoantibodies against platelet membrane antigen, mostly glycoprotein complexes IIb/IIIa [1]. In most cases, ITP develops after a viral infection [4].

ITP manifests with sudden purpura and hematoma in previously healthy children. Skin changes such as petechiae and purpura can be found in 60% of children, whereas mucous membrane bleeding (epistaxis, buccal bleeding, hematuria, menorrhagia, and gastrointestinal bleeding) can be found in 40% of children [9,10]. Approximately 3% of children develop severe bleeding events [11]. The most severe complication of ITP is intracranial bleeding, with an incidence of 0.1%–0.8%, which is more often found in children with a platelet count under 10 × 10^9^/L [1,11,12,13].

Before starting medical treatment, the intensity of the disease should be determined. Clinical examination is more important than the number of platelets because children with severe thrombocytopenia (˂10 × 10^9^/L) may have mild symptoms [14].

Various bleeding scores are available for the assessment of bleeding severity in ITP patients. They are used to standardize the treatment choice as well as follow up on the administered therapy [8,15,16,17,18]. In our study, we have chosen two recent scores, an ITP Bleeding Scale (IBLS) assessment system [17], and skin-mucosa-organ-gradation (SMOG) of bleeding score [18], which will be detailed in the Materials and Methods section.

The initial approach to ITP patient treatment is often an individual one. Generally, a “watch and wait” approach is used and later followed up with or without pharmacological treatment [19,20,21]. Approximately 50%–70% of children recover spontaneously within 3 months. Although the “watch and wait” approach is effective in the majority of children [2,22], better quality of life and fewer relapses were found in those with pharmacological intervention [23,24,25,26]. Commonly used medications include intravenous immunoglobulin (IVIG), intravenous anti-D immunoglobulin (anti-D) or glucocorticoids and, recently, agonists of thrombopoetin receptors (TPO-R) [1,27].

Vitamin D (VD) is a pro-hormone produced from 7-dehydrocholesterol when exposed to ultraviolet light radiation. It is biologically inert and to be activated, it has to be metabolized in the liver into 25-hydroxyvitamin D (25(OH)D), and then in the kidneys into 1α, 25-hydroxyvitamin D3. The active form of VD affects the absorbance of calcium and phosphate in the intestine, calcium mobilization in bones and reabsorbance of calcium in the kidneys [28]. VD function is not only limited to its role in the homeostasis of calcium, its action is additionally mediated via the VD nuclear receptor (VDR), which belongs to the superfamily of steroid hormone receptors and thyroid gland hormones [29,30].

In recent years, the importance of VD has been examined in immune and inflammatory response. VD regulates hormone secretion and cell proliferation and differentiation [31,32]. VD is also the regulator of the hematopoietic system—it modulates the activation and proliferation of lymphocytes, induces differentiation of monocytes in the promyelocytes and inhibits the secretion of several cytokines in T-cells [33,34].

Various studies have demonstrated the ability of VD to suppress the synthesis of interferon-gamma (IFNγ) and interleukin-2 (IL-2) in peripheral blood lymphocytes (PBL) and T-cell lines [35,36], confirming its immune-modulating function. Correlation between hypovitaminosis D and a higher incidence of infections and autoimmune diseases was reported as well [37,38,39,40,41,42]. It has been demonstrated that supplementation with VD is beneficial for Th1-mediated autoimmune response [43]. Likewise, VD insufficiency turns immune response towards a loss of tolerance [42,44].

Fatizzo et al. found low 25(OH)D values in adults with ITP [45], but further studies on this matter are warranted. To the best of our knowledge, there are no studies about the influence of hypovitaminosis D on ITP clinical presentation in children, especially studies that used different bleeding assessment tools. With this study, we analyze whether hypovitaminosis D is associated with a lower platelet count and severity of the clinical presentation of ITP in children.

Our hypothesis is that children with ITP who have lower levels of 25(OH)D at the time of ITP diagnosis will have more pronounced bleeding (indicated through higher values of bleeding SMOG and IBLS scores) in addition to a lower platelet count in peripheral blood.

## 2. Materials and Methods

### 2.1. Study Design

The study was an observational cross-sectional study.

Our primary outcomes were the correlation between 25(OH)D value and platelet count and the correlation between 25(OH)D value and clinical presentation of ITP determined using SMOG and IBLS bleeding scores at the time of ITP diagnosis.

Our secondary outcomes were the correlation between the patient gender and age with the severity of ITP. We also evaluated the correlation of the same variables with 25(OH)D values.

The Ethics Committee of the University Hospital of Split and Rijeka has approved this study. Written informed consent has been signed by all the participants’ parents.

STrengthening the Reporting of OBservational studies in Epidemiology (STROBE) guidelines were used for the creation of this paper [46].

### 2.2. Study Population

Participants were children who were hospitalized in Departments of Pediatrics (Division for pediatric hematooncology) due to newly diagnosed ITP at the University Hospital of Split in the period from 1 January 2013 until 31 December 2018 and at the University Hospital of Rijeka in the period from 1 January 2017 until 31 December 2018. We included eligible participants consecutively. Inclusion criteria were age between 0 and 18, ITP diagnosed by clinical examination and laboratory workup (platelet count in peripheral blood < 100 G/L). Patients whose parents did not consent to participation in the research and patients with the missing data were excluded. We also excluded patients who received blood products in the period of one month before the diagnosis of ITP as well as patients with secondary thrombocytopenia as part of another confirmed acute and/or chronic disease which was not ITP.

### 2.3. Variables

For every participant, after being hospitalized for the first time due to ITP, the following parameters were evaluated: 25(OH)D value, platelet count in peripheral blood, SMOG score, and IBLS score. We also collected data on patients’ age, gender, and place of residence on the day of ITP diagnosis. Medical records were used to collect mentioned data.

Patients’ peripheral venous blood was sampled in a standard BD Vacutainer^®^ SSTII Advance (BD, Plymouth, UK) tubes and sent to Department of medical laboratory diagnostics of University Hospital of Split and Rijeka for analysis. The level of serum 25(OH)D was analyzed by using commercially available Elecsys Vitamin D total assay with a Cobase601 analyzer (Roche Diagnostics International Ltd., Rotkreuz, Switzerland). This essay measures 25(OH)D values using a competitive electrochemiluminescence binding technique. The detection range of the test is 7.5–175 nmol/L 25(OH)D, and the sensitivity of the assay is 5 nmol 25(OH)D/L. The intraclass coefficient of variations (CV) was 5.3% at 39 nmol 25(OH)D/L, 5.6% at 67 nmol 25(OH)D/L, 6.7% at 165 nmol 25(OH)D/L, 2.2% at 174 nmol 25(OH)D/L, 3.9% at 71.8 nmol 25(OH)D/L, and 5.2% at 39.5 nmol 25(OH)D/L. In addition, the Limit of Blank, Limit of Detection, and Limit of Quantification were 5.0, 7.5 and 12.5 nmol/L, respectively. All the samples were analyzed in duplicate. The method was standardized based on international standards. Sufficient and recommended 25(OH)D values in children are >75 nmol/L, and everything below that level is regarded as hypovitaminosis D [47,48]. If 25(OH)D value was between 50 and 75 nmol/L, it was considered as insufficiency, while 25(OH)D values under 50 nmol/L were considered VD deficiency [48].

At the time of diagnosis, patients were examined to assess the severity of clinical presentation by calculating the bleeding score according to the recommendations of the International Working Group (IWG) for ITP in the SMOG system [18] and also the IBLS system that was created by Page and Psaila [17]. Each bleeding score was calculated by two independent pediatric hematogists. In the case of a difference between the scores, the scores were checked by a head of division for pediatric hematooncology.

The SMOG system is a system for the categorization of bleeding level in ITP. The score of bleeding according to SMOG system is calculated as follows: bleedings are grouped into 3 main domains: S (skin), M (mucous membranes), O (organs) with gradation of the intensity of bleeding (G). Ratings are given at the time of examination from 0 up to 3 or 4 (with a score of 5 for fatal bleeding). For mucous membranes (e.g., epistaxis), the rating scale is from 0 to 4; for the organ domain (except for intracranial and intraocular bleeding), the rating scale is 0, 2, 3 and 4. For other places of bleeding (in skin and mucous membranes), ratings of 0 to 3 are assigned. If the patient reports bleeding but there is no evidence for that during examination or in medical documentation, a score of 1 is given. Within each domain, the same rating is given for bleedings with similar clinical influence. For the exact evaluation of bleeding in each domain, a table is used for the assessment, which can be found on the official website of IWG. For example, if the rating for a skin domain is 2, for mucous membranes 1 and organs 1, then the final index is S2M1O1. It can also be used to sum up mentioned ratings according to domains to obtain a final numerical rating. A higher SMOG score represents more severe bleeding [18]. In this study, we used SMOG final numerical ratings and individual ratings in skin, mucosa and organs for statistical analysis.

The IBLS system assigns ratings from 0–2 for 9 selected sites of bleeding (skin, mouth, epistaxis, gastrointestinal, urinary, gynaecological, pulmonary, intracranial, and subconjuctival) [17].

### 2.4. Statistics

#### 2.4.1. Calculation of the Minimal Sample Size

To prove our first hypothesis, we used unpublished and published data from a pilot study of Culic et al. [49] for calculating our minimal sample size. In that pilot study, 21 participants were included. For the modeling of correlation between the 25(OH)D and SMOG score, we used a linear model and Deming’s methodology [50]. Based on the data from the pilot study, even with 21 participants, we achieved statistical relevance for the slope (−0.06, 95% confidence interval (CI) (−0.22 to −0.05)). However, we need to increase the number of participants to a minimum of 30 to keep the power of the study at 80% and the probability of type 1 error at 5%.

Deming’s methodology is a method of linear regression type 2 (Method II), using a Reduced Major Axis (RMA) variant [50]. In our pilot study [49], we obtained results that the 95% CI coefficient slope does not include 0, according to which we already have enough patients not to commit a type 2 error; so, our only concern is to keep the formal level of type 1 error at 5%. As there is no software to calculate the size of sample for the RMA variant of regression, we used network software to calculate the sample size (Soper DS, a priori sample size calculator for multiple regression, 2017) and concluded that the sample we can muster (30 patients) guarantees a margin of type 1 error of 5% and power of 80% to discover medium to large (Cohen *f*^2^ = 0.29) differences between the null and alternative hypotheses.

#### 2.4.2. Statistical Tests Used in the Study

Depending on distribution, descriptive data were shown as a mean value with standard deviation. The correlation between SMOG score with 25(OH)D values was examined by Spearman’s correlation coefficient and linear regression. Furthermore, relationships between the components of SMOG score and 25(OH)D values were tested by a test for linear trend or t test. Statistical analysis was performed in GraphPad Software (La Jolla, CA, USA), and the level of significance was set to 0.05. For each effect size, a 95% CI was given.

## 3. Results

During the study period, a total of 67 newly diagnosed patients with immune thrombocytopenia—57 from University Hospital of Split and 10 from the University Hospital of Rijeka—were eligible for inclusion in the study. Of these, 22 subjects (32%) were excluded due to: diagnosis of chronic thrombocytopenia (11), secondary thrombocytopenia as part of another confirmed acute and/or chronic disease (3), and missing data (8). In total, 45 patients (67%) were included in the study.

There were 22 males (48%) and 23 females (52%). VD insufficiency was detected in 20 patients (44%), VD deficiency in 10 patients (22%), and 15 patients (33%) had a sufficient value of 25(OH)D at ITP diagnosis.

Distribution data of the age at presentation, platelet count, 25(OH)D value, SMOG and IBLS score can be found in Table 1.

In our study, 28% of patients (13) had more severe mucosal or organ bleeding (in SMOG M or O value > 2) on the day of ITP diagnosis. Of those 13 patients, six had mucosal bleeding, seven had organ bleeding and four had both mucosal and organ bleeding. There were no cases of intracranial bleeding in our study.

### 3.1. Correlation between 25(OH)D and SMOG Score

**Figure 1:**
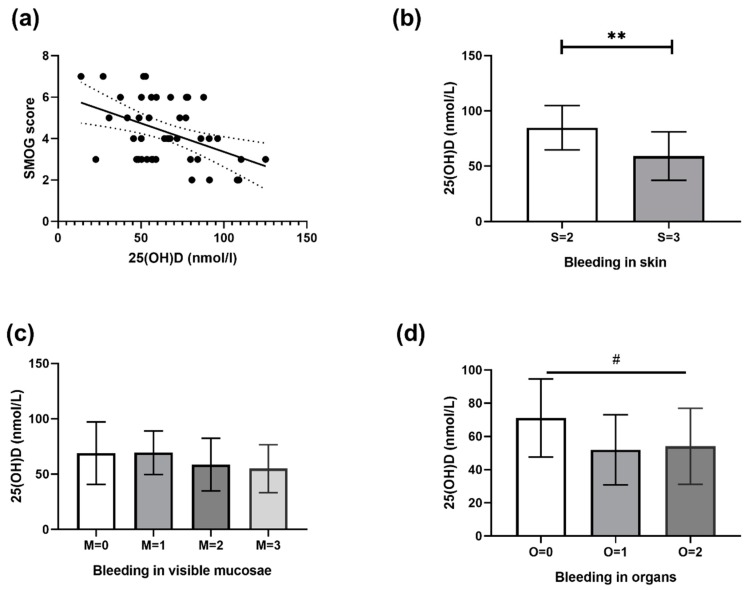
(**a**) Graph of linear regression between 25(OH)D value and skin-mucosa-organ-gradation (SMOG) bleeding score. The mean slope was −0.027, with the intercept at 5.99, while the 95% confidence interval (CI) was at −0.04 to −0.007 (intercept 4.6 to 7.3). *R*^2^ = 0.15, *p* = 0.006. (**b**) Difference between mean 25(OH)D values of two groups of patients with bleeding in skin (S) ratings 2 and 3 calculated with SMOG score (*t* test, *p* = 0.001, 95% CI 10.5 to 40.5). (**c**) Distribution of mean 25(OH)D values in groups of patients with a different severity of bleeding in mucosae using the linear trend test. (**d**) Distribution of mean 25(OH)D values in groups of patients with a different severity of bleeding in organs (calculated with SMOG score) using the linear trend test (slope −9.55, 95% CI −0.54 to −18.56, and *p* = 0.03). *R*^2^ coefficient of determination, ** 0.001 ≤ *p* < 0.01 for *t* test, # 0.01 < *p* < 0.05 for test for linear trend. S: Skin, M: Mucous membranes, O: Organs

We found a significant linear relationship between 25(OH)D value and SMOG (*p* = 0.006), which shows that patients with a lower 25(OH)D value have a higher SMOG score. To further confirm that hypovitaminosis D inversely correlates with the SMOG score, we also compared 25(OH)D values with every main domain of SMOG score. In patients with a different severity of bleeding in the skin (S parameter in SMOG score), we found that patients with a lower 25(OH)D value had more severe bleeding in the skin with a difference between means (S2 and S3 group in SMOG score) of 26 ± 7 nmol/L (*p* = 0.001). There was no significant connection between the severity of bleeding in mucosa and 25(OH)D values. We found a significant negative trend (*p* = 0.03) that showed us there is more severe organ bleeding in patients with lower 25(OH)D values. These results are all shown in detail in Figure 1.

Using the Spearman nonparametric correlation, we also found a statistically significant correlation between the 25(OH)D value and the SMOG score (*p* = 0.01, Spearman *r* = 0.36).

### 3.2. Correlation between 25(OH)D and IBLS Score

**Figure 2:**
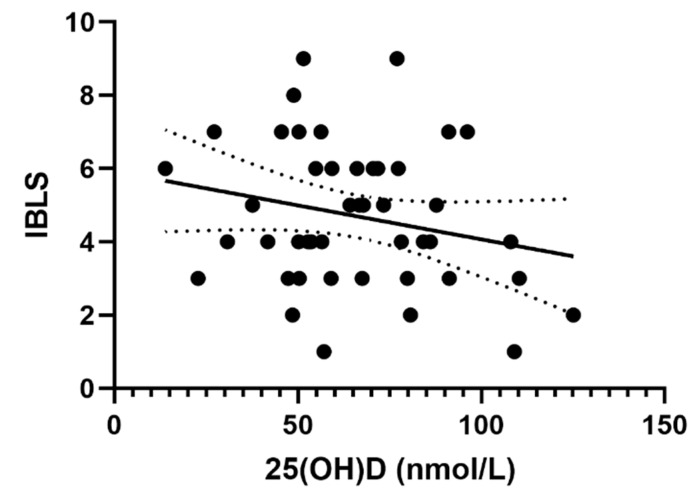
Graph of linear regression between Vitamin D (VD) value and ITP Bleeding Scale (IBLS) score. The mean slope was −0.018, with the intercept at 5.92, while the 95% CI was at −0.04 to −0.005 (intercept 4.2 to 7.6). *R*^2^ = 0.05, *p* = 0.13.

When we analyzed the VD value and IBLS bleeding score, we found a negative correlation between the two variables but it was not significant (*p* = 0.13) (Figure 2). With Spearman nonparametric correlation, we obtained similar results (Spearman *r* = −0.18, *p* = 0.22).

### 3.3. Correlation between 25(OH)D and Platelet Count

There was no significant correlation between the 25(OH)D value and the platelet count at the time of diagnosis in the linear regression (*R*^2^ = 0.0001, *p* = 0.93) or in the Spearman nonparametric correlation (Spearman *r* = −0.06, *p* = 0.67).

### 3.4. Correlation between Patient Gender and Age with 25(OH)D and SMOG Score

As our secondary outcome, we evaluated the connection between patient gender and age with the severity of ITP and those same variables with hypovitaminosis D.

We found a positive correlation between patients’ age and SMOG score (Spearman *r* = 0.33, *p* = 0.02) i.e., older patients have higher SMOG score and therefore more severe bleeding. There was no difference in SMOG score between genders (similar median SMOG score for both genders, Mann Whitney test *p* = 0.15).

We also found negative correlation between patients’ age and 25(OH)D values (Pearson *r* = −0.42, *p* = 0.003), i.e., older patients have lower 25(OH)D values. There was no difference in 25(OH)D values between genders (the median 25(OH)D value for males was 70 ± 25, and for females 62 ± 22, *p* = 0.25).

## 4. Discussion

There are some important findings in this study that will be subsequently discussed. First, patients with lower 25(OH)D values have more severe bleeding measured with the SMOG bleeding score at ITP diagnosis. This confirms our initial hypothesis that more severe hypovitaminosis D at the time of ITP diagnosis in children is associated with more severe bleeding. Secondly, the results showed that majority of patients with newly diagnosed ITP have hypovitaminosis D. However, we did not find a correlation between 25(OH)D values and platelet count at ITP diagnosis.

### 4.1. Demographics and Clinical Presentation of ITP

The patients analyzed in our study are similar and comparable with other patients in pediatric studies on ITP [2,51,52]. Severe bleeding was found in 28% of patients (*n* = 13) on the day of ITP diagnosis, which is slightly higher than the incidence reported in a large systematic review [13]. However, we had no patients with intracranial bleeding. That is the most severe complication of ITP and is relatively uncommon (0.4% of children) [13].

Until recently, the main marker for the severity of ITP was platelet count but it is concluded that clinical presentation, primarily bleeding events, was more significant in the assessment of ITP severity [6,14]. Therefore, ITP is considered severe only in patients who have clinically relevant bleeding and require additional therapeutic intervention or a change in the active treatment of ITP i.e., increased dose [6].

Recent studies also outline a problem in ITP bleeding severity quantification. Although two bleeding assessment tools that we used in our study (SMOG and IBLS) proved to be useful for the gradation of bleeding severity [13,53,54], we still agree that a uniform high-quality bleeding assessment tool is needed for bleeding assessment in ITP.

### 4.2. Correlation between Hypovitaminosis D and ITP

The mean 25(OH)D value at ITP diagnosis in our study (65 ± 24 nmol/L) can be considered as hypovitaminosis D [47,48,55]. Liu et al. reported a mean 25(OH)D value of 43 ± 13 nmol/L in adult patients with ITP, while Yesil et al. reported a mean 25(OH)D value of 47 ± 21 nmol/L in children with ITP [56,57]. The higher values in our study might be explained with the difference in the geographic origin of the patients that were included in our study. Our patients come from the coastal Croatia region, with a high incidence of sunny days [58], and therefore they may have higher 25(OH)D values. The study by Culic et al. had pediatric patients from a similar geographic region and also had lower 25(OH)D values, especially in the group of patients with chronic ITP (35 ± 20 nmol/L) probably due to ITP treatment which additionally lowered their 25(OH)D values [49].

Similar to our study, a high incidence (up to 80%) of hypovitaminosis D was previously reported in children with ITP [59]. Fattizzo et al., who evaluated adult patients with autoimmune cytopenias including ITP, suggested that VD influences the immune system by favorizing Th1 immune response and therefore lowers the chance for the development of autoimmune cytopenias including ITP [45].

To the best of our knowledge there are not many studies that connect ITP and hypovitaminosis D. But there are lots of studies connecting hypovitaminosis D with other autoimmune diseases, confirming the immunomodulatory influence of VD. The high incidence of hypovitaminosis D was found in patients with diabetes mellitus type 1 [60], autoimmune thyroid gland disease [61,62], multiple sclerosis [63], systemic lupus erythematosus [64,65], rheumatoid arthritis [66,67,68], and Crohn’s disease [69]. A large well-designed study by Skaaby et al. [70] found significant inverse associations between vitamin D status and the development of autoimmune diseases. Bizzaro et al. [55] also confirmed that 25(OH)D values and VDR polymorphism are connected with various autoimmune diseases. As ITP is an autoimmune disease with a similar pathophysiological mechanism, we can assume there is a similar connection between VD and ITP.

Some authors even took a step forward and administered VD as a therapy in autoimmune diseases such as rheumatoid arthritis [71] and psoriatic arthritis [72] with promising results. With regard to ITP, Bockow and Kaplan described two cases of refractory ITP that were successfully treated with high-dose VD supplementation and hydroxychloroquine [73]. In several studies where VD supplementation therapy was implemented, the bottom limit of 25(OH)D value, at which the therapy started, was 50 nmol/L [60,61] and 75 nmol/L [73]. The British Society for Haematology recently also recommended VD supplementation in patients with ITP as a prevention for glucocorticoid-induced osteoporosis [74].

Based on our results, we can also recommend VD supplementation in patients with newly diagnosed ITP who have hypovitaminosis D.

### 4.3. Correlation between VD and ITP Clinical Presentation

In this study, we measured three indicators of the clinical severity of ITP at the time of the initial diagnosis: platelet count and two bleeding scores (IBLS and SMOG). Using linear regression and nonparametric correlation, we found a significant negative correlation between 25(OH)D value and SMOG score. We found a negative correlation between 25(OH)D value and IBLS score, but it was not significant. There was no significant correlation between 25(OH)D and platelet count as a third evaluated indicator of clinical severity. Some authors suggest that platelet count is not a quality marker of the clinical severity of ITP at all [6], and so that might partially explain this negative result.

To further investigate the correlation between SMOG score and 25(OH)D, we evaluated each part of the SMOG score and found a significant negative correlation between 25(OH)D values and bleeding in the skin and the organ. In our opinion, the description of each type of bleeding in the SMOG system is more detailed, producing a more accurate final score, but it is time consuming and can be a problem in daily clinical practice.

To the best of our knowledge, there are no studies connecting 25(OH)D values and the clinical presentation of ITP in this manner. Fattizzo et al. found a correlation between VD levels and disease severity at the onset of ITP and other autoimmune cytopenias in adults [45], but they did not use bleeding assessment tools to assess the clinical presentation of ITP. Some authors that were mentioned before also found a connection between 25(OH)D values and the activity or severity of some autoimmune diseases [64,67,69]. Based on those results and the connection between VD and the incidence or activity of the autoimmune diseases, VD has been suggested as a possible therapy for them [64,65,75]. Antico et al. speculated that VD at high doses can affect the development or even the symptoms of the autoimmune diseases [40]. Accordingly, some of the authors suggested VD as a new therapy option for the treatment of ITP [45,49,57]. However, most of the mentioned authors agree that more studies, especially randomized clinical trials, are needed to further evaluate the influence of VD on autoimmune diseases.

## 5. Limitations

In this study, we only recruited patients from the coastal part of Croatia, and so there is a chance of selection bias based on the geographical origin of the patients. It is possible that patients who live in southern Croatia have higher sun exposure than in other geographic region [58] and therefore could have higher average 25(OH)D values than patients in other studies.

A second bias could be information bias. SMOG and IBLS bleeding scores are calculated from the clinical signs of bleeding. Pediatric hematologists described those signs during the clinical examination of the patient. Since the patient can be examined by a number of hematologists, there is a possibility of some form of information bias due to the subjective interpretation of the clinical finding. With standardization of the clinical examination of our patients and with good planning, we attempted to prevent this kind of bias.

## 6. Conclusions

Hypovitaminosis D is very common in children with ITP, as in other autoimmune diseases. Hypovitaminosis D influences the severity of ITP in children at the initial diagnosis of the disease and therapy with VD could be a new potential option for the treatment of ITP. To investigate the connection between 25(OH)D and the incidence and severity of ITP, further studies, especially randomized controlled studies, are needed.

## Figures and Tables

**Table 1:** Distribution of age, 25(OH)D, platelet count, IBLS and SMOG score at presentation of ITP (*n* = 45).

Variable	All Patients (*n* = 45)	Sufficient 25(OH)D Value (*n* = 15)	Hypovitaminosis D (*n* = 30)
	**Mean ± SD**		
Age at presentation, year	5.6 ± 4.8	3 ± 3.3	6.9 ± 5
25(OH)D, nmol/l	65 ± 24	93 ± 15	52 ± 15
Platelets, G/L	9.8 ± 10	6.9 ± 7.7	11 ± 11
IBLS ^1^	4.7± 1.9	4.2 ± 2.1	4.9 ± 1.8
SMOG ^2^	4.3 ± 1.5	3.6 ± 1.4	4.6 ± 1.5

^1^ IBLS—Immune thrombocytopenia bleeding scale [17]; ^2^ SMOG—skin-mucosa-organ-gradation of bleeding in immune thrombocytopenia [18]; SD—standard deviation.
